# Current insights into insect immune memory

**DOI:** 10.7554/eLife.105011

**Published:** 2025-07-01

**Authors:** Gabriela Krejčová, Adam Bajgar

**Affiliations:** 1 Department of Molecular Biology and Genetics, Faculty of Science, University of South Bohemia České Budějovice Czech Republic; Münster University Hospital Münster Germany; Yale University New Haven United States

**Keywords:** immune memory, trained immunity, insect immunity, *Dscam*, hemocytes, insect vaccination

## Abstract

Traditionally, insects have been thought to be entirely dependent on their innate immune system, which has little capacity for the acquisition of experience from previous infections. However, much experimental evidence has challenged this view, showing that insects can develop long-term, pathogen-specific immune memory, which in some cases can be transmitted to offspring. Although significant progress has been made in this area, the underlying mechanism is still not fully understood, and a number of fundamental questions remain unanswered. In this review, we present an overview of documented cases of insect immune memory and summarize the experimental evidence in support of the prevailing hypotheses on the mechanism of antiviral and antibacterial immune memory in insects. We also highlight key questions that remain unanswered and discuss *Drosophila melanogaster* as a powerful model organism for investigating the mechanisms of innate immune memory formation. Finally, we evaluate the significance of this research and explore the potential for insect vaccination.

## Acquired immunity: More prevalent than we thought

Virtually all living organisms possess some form of immune system, which ensures the recognition of self from non-self and protects the integrity of the organism and its genetic information. Individual immune mechanisms have evolved over millions of years of coevolution between the host’s immune system and the pathogens attempting to evade it (Müller et al., 2018). Acquisition of experience from the initial contact with the pathogen, therefore, represents the crucial advantage for hosts that can gain resistance to reinfections (Palmieri et al., 2021).

**Figure 1.**
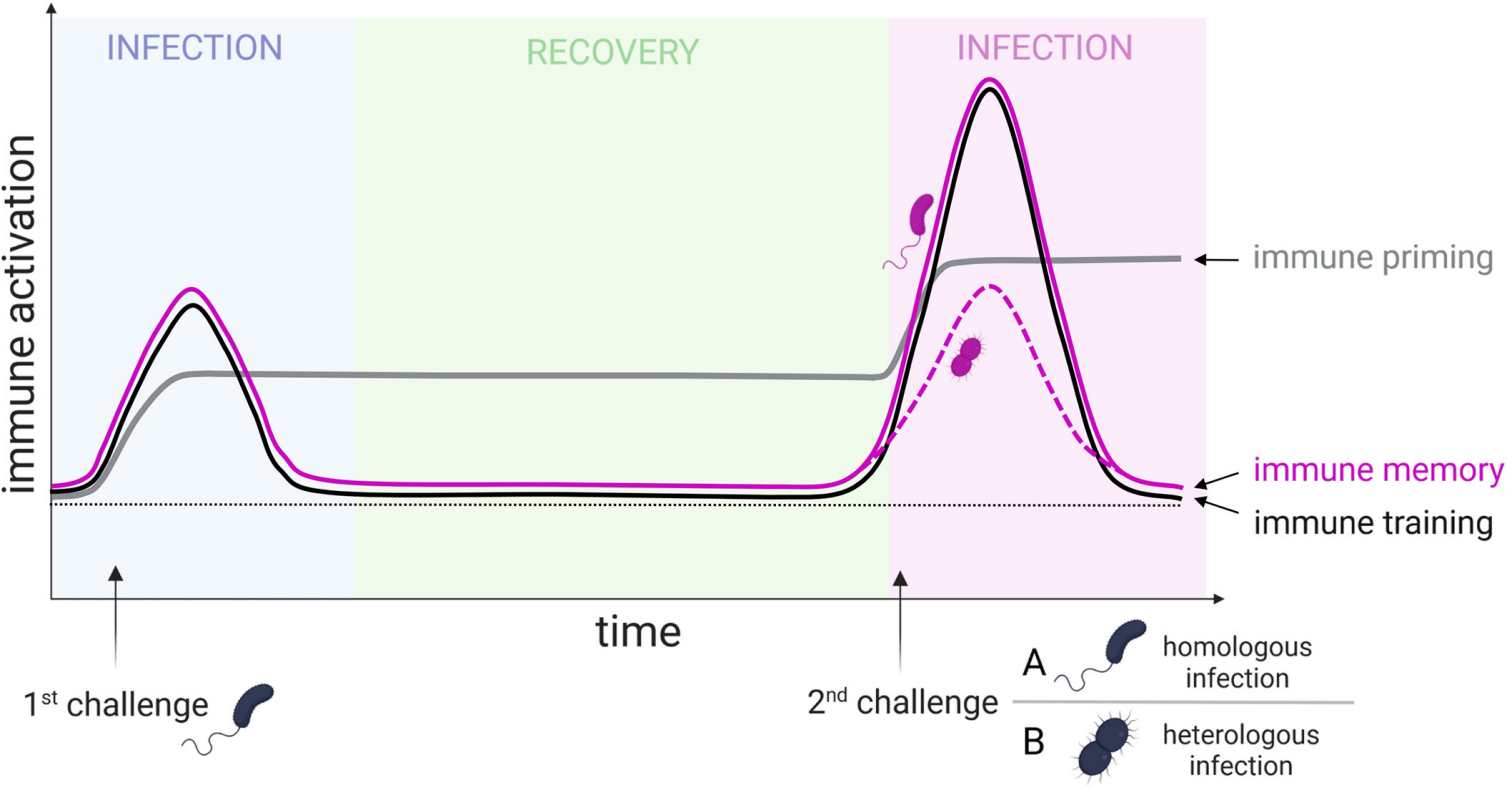
Graphical representation of the progression of acquired immunity in insects (immune priming, immune training, and immune memory). Immune priming is characterized by the persistent activation of the immune system following the first immune challenge, resulting in an enhanced immune response to the second challenge. In contrast, immune training and immune memory are biphasic, meaning immune response returns to baseline before the second challenge. While immune priming and immune training are pathogen-nonspecific responses, immune memory operates in a pathogen-specific manner, i.e., the same pathogen must be involved in both the first and second challenges (homologous infection; solid line). In the case of heterologous infection (where different pathogens are involved in the first and second challenges), immune memory is not induced (dashed line). The horizontal black dotted line indicates the immune response baseline. This figure was created using BioRender.com.

In nature, we can observe various sophisticated strategies that allow organisms to gain a certain degree of immune experience at both the systemic and cell-autonomous levels (Divangahi et al., 2021). While the term ‘*acquired immunity’* was previously used almost exclusively to refer to adaptive immunity in jawed vertebrates, it is now applied more broadly to encompass various forms of acquired immune experience (Little et al., 2005; Rimer et al., 2014; Cooper and Eleftherianos, 2017). Currently, three basic categories of acquired immune responses (immune priming, immune training, and immune memory; Box 1) are distinguished according to the character and specificity of the initial immune response (Tang et al., 2023; Vuscan et al., 2024; Netea et al., 2019; Figure 1). The original anthropocentric belief that only vertebrates with adaptive immune systems can form specific and long-lasting immune memory is incorrect. Advances over the last two decades in comparative and evolutionary immunology have revealed complex mechanisms underlying the adaptability of immune response in prokaryotic bacteria and archaea, and various degrees of acquired immunity can be observed in almost all eukaryotes (Newsom et al., 2020; Bagasra and Prilliman, 2004; Gomes et al., 2022).

Box 1. **Immune priming:** Following the first challenge, the immune activation remains elevated and does not return to baseline, enhancing resistance to secondary infections through nonspecific immune mechanisms.

**Immune training:** The primary challenge induces long-term changes in the immune system, leading to nonspecific sensitization for the secondary activation. Immune training is biphasic, with common immune response readouts returning to baseline between stimuli.

**Immune memory:** The primary challenge with a specific pathogen induces long-term changes in the immune system, allowing for faster and stronger pathogen-specific recognition and elimination upon a secondary challenge with the same pathogen. Immune memory is biphasic, with common immune response readouts returning to baseline between the stimuli.

Whole-genome sequencing and comparative analysis of hundreds of prokaryotic organisms have led to the discovery of genomic islands that accumulate immune-related genes, enabling the identification of more than 150 distinct prokaryotic immune mechanisms (Bernheim et al., 2024). Notably, many of these mechanisms are also present to some extent in eukaryotes, indicating that particularly cell-autonomous immune mechanisms are remarkably conserved across the tree of life (Mayo-Muñoz et al., 2023). The prevailing hypothesis for this phenomenon is that immune mechanisms that evolved during the rapid evolution of prokaryotes, as protection against mobile genetic elements and other pathogens, were later adopted and repurposed by eukaryotic immune systems. The acquisition of these immune traits likely occurred through horizontal gene transfer mediated by retrotransposons, highlighting the central role of domesticated mobile genetic elements in the evolution of the eukaryotic immune system (Wein and Sorek, 2022).

A particularly interesting example of an adaptive immune mechanism in prokaryotes is the Clustered Regularly Interspaced Short Palindromic Repeats (CRISPR)-Cas system. This system is widely present in most archaea and many bacteria, where it serves as a defense mechanism against bacteriophages (Rath et al., 2015). When a bacterium or archaeon is infected by a bacteriophage, it can recognize the foreign nucleic acids, cleave them into smaller fragments, and integrate these fragments into its own genome. Viral DNA fragments are inserted into a specific region of the host genome known as CRISPR, which acts as a repository of previous viral encounters (Wang et al., 2022).

Upon subsequent viral infection, the CRISPR locus is transcribed, producing CRISPR-pre-mRNA, which is then cleaved into individual CRISPR RNAs. These short RNA fragments guide Cas nucleases to perform sequence-specific cleavage of viral genetic material (Hille et al., 2018). This sophisticated mechanism is widely regarded as a clear example of adaptive immunity since the integration of viral sequences into the host genome serves as a form of immune memory, while sequence-specific cleavage ensures the specificity of the immune response (Jiang and Doudna, 2017). Interestingly, key enzymes that protect prokaryotes from mobile genetic elements are co-opted transposases derived from retrotransposons (Koonin and Krupovic, 2015).

A similar phenomenon is observed in the adaptive immune system of jawed vertebrates, where the enzymes responsible for somatic recombination (*Rag1* and *Rag2*) in T and B lymphocytes are derived from the Transib family of retrotransposons (Huang et al., 2016). Another example, which will be discussed in detail later in this review, is the adaptive antiviral immunity in insects. In this system, endogenous transposons Ty1 and Ty3 play a central role in acquiring copies of viral nucleic acids and integrating them into the genome, thus creating a long-lasting and heritable immune memory (Saleh et al., 2009; Tassetto et al., 2017). These examples reveal intriguing parallels in the convergent evolution of mechanisms of acquired immunity (Eaglesham and Kranzusch, 2024), all of which exploit the enzymatic activities of genes acquired from domesticated retrotransposons to establish immune memory by modifying host genomic information. Comparative and evolutionary immunology may offer valuable insights into the discovery of as-yet-unidentified mechanisms of immune memory.

### Insect immune system

Insects are the most abundant group of animals on Earth (Stork, 2018), and like other animals, they are exposed to a wide range of pathogens throughout their lifetimes, including viruses, bacteria, fungi, protozoa, nematodes, and parasitoids (Federici, 2009). While certain pathogens can cause significant declines in entire insect populations, others are tolerated to varying degrees. In such cases, insects can act as reservoirs for these pathogens, serving as vectors that facilitate their amplification and transmission, including to humans (St Leger, 2021).

**Figure 2.**
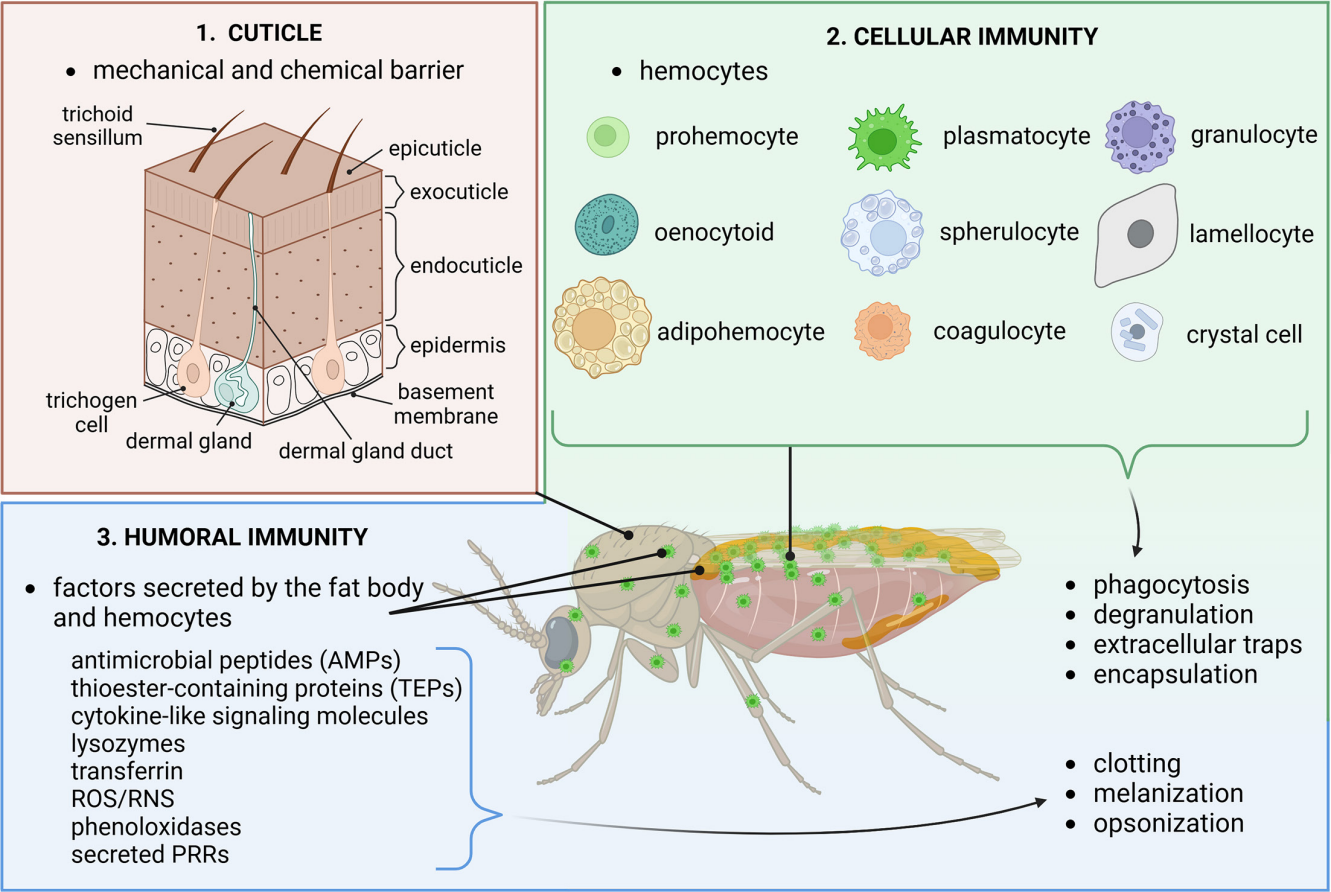
Overview of the insect immune system. The cuticle forms the first immune barrier, protecting insects from most pathogens in their environment. However, if this barrier is breached, the immune response is activated, consisting of a coordinated reaction between cellular and humoral immunity. The cellular immune response is mediated by several types of immune cells collectively known as hemocytes, which perform specialized immune functions such as phagocytosis, degranulation, extracellular trap formation, and encapsulation. The humoral immune response involves a diverse array of secreted factors that directly eliminate pathogens in circulation. These factors are produced not only by hemocytes but also by the fat body, an immunometabolic tissue. Circulating immune factors contribute to hemolymph clotting, melanization, and pathogen opsonization. Through the combination of these mechanisms, insects can effectively defend against a wide range of pathogens, including bacteria, fungi, viruses, parasitic nematodes, parasitoids, and protozoa. This figure was created using BioRender.com.

In insects, the primary immune defense is provided by the cuticle, which acts as a physical barrier, offering strong protection against most pathogens in the environment. Consequently, the most common routes of infection are through wounds, the digestive system, or the tracheal system. Once the cuticle is breached, immune cells are activated, triggering both cellular and humoral immune responses (Buchon et al., 2014; Figure 2).

The insect innate immune system is largely based on evolutionarily conserved pathways, which are also found, to some extent, in mammals. The cellular branch includes various effector immune cells, known as hemocytes, which perform distinct functions during the immune response, such as phagocytosis (plasmatocytes), encapsulation (lamellocytes), activation of phenoloxidases (crystal cells), degranulation (granulocytes), or the release of extracellular traps (Mahanta et al., 2023). In addition, some insect species possess specialized immune cell types, such as spherulocytes, oenocytoids, coagulocytes, adipohemocytes, and thrombocytoids, which further contribute to pathogen elimination, wound healing, metabolic homeostasis, as well as nutrient transport (Eleftherianos et al., 2021). Though not present in all insect species, an alternate approach to pathogen neutralization is the formation of extracellular traps. These extracellular web-like structures, composed of double-stranded DNA, histones, elastase, and myeloperoxidase, serve to trap, immobilize, and kill pathogens (Nascimento et al., 2018). Although insect immune cell types and their functions are less diverse than those in vertebrates, the key signaling pathways and transcription factors involved in phagocytosis (Melcarne et al., 2019), hematopoiesis (Banerjee et al., 2019), cell proliferation, and differentiation (Evans et al., 2003) have remained conserved for over 550 million years of evolution.

Pathogens are detected by pathogen recognition receptors that can be classified into several groups, such as NOD-like receptors, C-type lectins, thioester-containing proteins, scavenger receptors, peptidoglycan recognition proteins, Toll-like receptors, or Gram-negative-binding proteins. These receptors activate general immune-related signaling pathways, such as Toll, IMD, *Janus kinase/signal transducers and activators of transcription* (JAK-STAT), or *Jun N-terminal kinase* (JNK). While Gram-positive bacteria and fungi predominately activate the Toll pathway, Gram-negative bacteria induce the IMD signaling pathway (Browne et al., 2013). Activation of cellular immunity is often associated with increased proliferation of immune cell precursors and their differentiation. These processes are coordinated by specific transcription factors and signaling pathways, including *erythroid transcription factors*, *vascular endothelial growth factor*, JAK-STAT, and JNK (Banerjee et al., 2019; Evans et al., 2003).

When encountering foreign objects too large for phagocytosis, such as parasitoid eggs or nematodes, insects utilize encapsulation and melanization (Bilandžija et al., 2017). The surface antigens of the parasitoid are recognized by macrophage-like plasmatocytes, which release signaling factors that trigger the extensive differentiation of progenitor prohemocytes and plasmatocytes into lamellocytes (Nakhleh et al., 2017). These large, sheet-like cells completely encase the foreign object in successive layers and secrete prophenoloxidases within the capsule. These enzymes catalyze the conversion of the amino acids phenylalanine and tyrosine into melanin, producing toxic free radical by-products that help eliminate the pathogen (Tang, 2009).

Insects constantly face an incredibly wide range of DNA and RNA viruses, and, therefore, it is not surprising that the antiviral response of insects is both complex and highly developed. The conserved intracellular immune response based on RNA interference (RNAi) remains the most thoroughly studied antiviral mechanism in insects (Gammon and Mello, 2015). In this pathway, viral double-stranded RNA is cleaved by the endoribonuclease *Dicer* into short small interfering RNA (siRNA) fragments. *Dicer*, together with the RNA-dependent nuclease *Argonaut*, forms the RNA-induced silencing complex (RISC), which facilitates the sequence-specific recognition and cleavage of viral nucleic acids (Leggewie and Schnettler, 2018).

Many immune and repair signaling pathways, such as Toll, IMD, and JAK-STAT, have also been implicated in antiviral defense. Activation of these pathways triggers a systemic antiviral response, which includes increased production of antimicrobial peptides, reactive oxygen species (ROS) and reactive nitric species, as well as the elimination of virus-infected cells through autophagy and efferocytosis by macrophage-like plasmatocytes (Kingsolver et al., 2013). While the interferon response characteristic of mammalian species has not been identified in insects, recent findings suggest that the cGAS-like receptor STING-Relish pathway plays a central role in general antiviral defense in *Drosophila*. In mammals, the cGAS-STING is a major component of the antiviral response to DNA viruses, leading to the activation of an interferon-based response. Similarly, in insects, it has been documented that STING signaling activates the IMD pathway transcription factor Relish to regulate the expression of genes potentially involved in antiviral defense (Goto et al., 2018; Decout et al., 2021).

The insect immune response is not solely dependent on the activity of the immune system itself, but requires coordinated adjustments across virtually all organs and tissues. Cytokines and metabokines produced by activated immune cells during an immune threat play a central role in orchestrating this coordination (Bajgar et al., 2021). Similar to mammalian macrophages, insect plasmatocytes undergo a fundamental metabolic rewiring in response to acute bacterial infection, shifting their predominant ATP metabolic pathway from oxidative phosphorylation to aerobic glycolysis (Krejčová et al., 2019). This metabolic adaptation provides sufficient energy and precursors for the production of ROS and other bactericidal effectors, though it also makes plasmatocytes highly dependent on external nutrient resources. To secure these resources, plasmatocytes secrete various factors, such as *Imaginal morphogenesis protein late 2*, to induce nutrient mobilization from the central metabolic organ while limiting their consumption by peripheral nonimmune tissues. Interestingly, this metabolic adaptation appears to involve the suppression of insulin signaling (Krejčová et al., 2023).

When introducing the insect immune system, the humoral branch cannot be omitted. It is responsible for producing a wide range of bactericidal substances, such as antimicrobial peptides, clotting factors, and signaling molecules. During systemic infection, antimicrobial peptides are rapidly expressed by immune cells and the fat body (Zhang et al., 2021). Due to their positive charge, these peptides act as opsonizing factors, embedding themselves in the hydrophobic portion of bacterial or fungal membrane. This facilitates recognition by immune cells, destabilizes the pathogen’s membrane, and ultimately leads to cell death (Dho et al., 2023).

Another common antimicrobial strategy in insects involves the coagulation of hemolymph, commonly referred to as clotting. Initially, the wound is covered by clotting proteins that are abundant in the hemolymph, such as thioester-containing proteins, integrins, lectins, lipoproteins, and storage peptides. These molecules are cross-linked by transglutaminase, which is homologous to factor XIIIa, a key component of the vertebrate coagulation cascade. As the process continues, the soft clot hardens through phenoloxidase-dependent cross-linking, resulting in hemolymph coagulation that restricts bacterial motility and division (Aprelev et al., 2019).

In addition to these intricate innate immune mechanisms, a growing body of evidence supports the existence of acquired immune mechanisms in insects. Although immune ‘adaptability’ in insects was not previously considered, presumably due to their shorter lifespan and thus the belief that they do not require immune memory, numerous documented cases of acquired immunity in insects now exist. While several insightful reviews have explored this topic in recent years (Cooper and Eleftherianos, 2017; Prakash and Khan, 2022), here we provide a systematic review of well-documented experimental evidence of acquired immunity in insects, with an emphasis on immune memory. Furthermore, we delve into the potential underlying mechanisms of antiviral and antibacterial immune memory and highlight unresolved questions that should be addressed in future research, possibly through the use of *Drosophila melanogaster*, which is exceptionally suited for genetic dissection of immune pathways. Finally, we discuss future directions for the field and explore how knowledge on innate immune memory mechanisms could inform the potential for insect vaccination.

## Acquired immunity in insects: From trained immunity to innate immune memory

**Figure 3.**
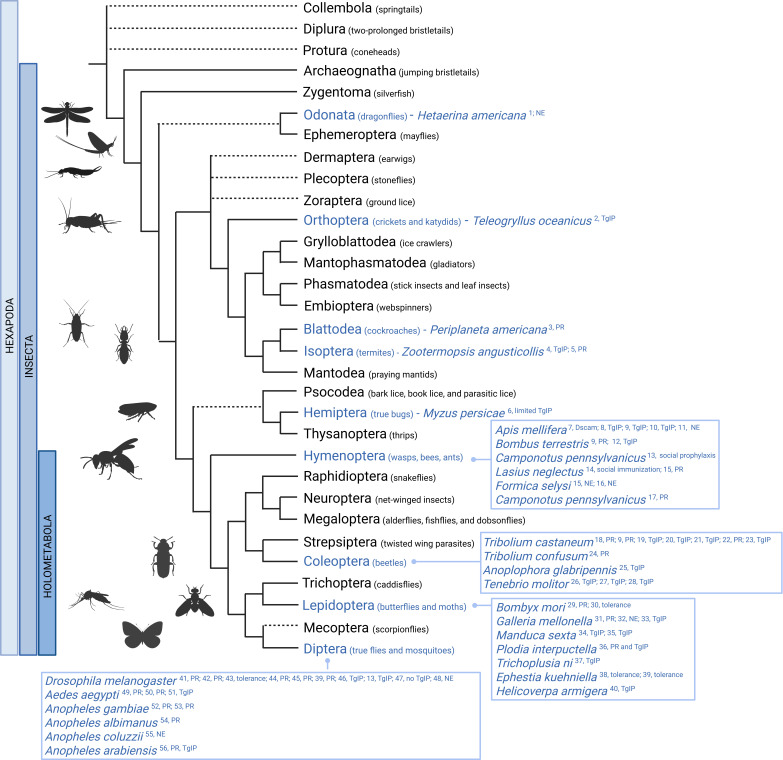
Simplified phylogenetic tree of insect orders with documented cases of acquired immune responses. Immune ‘adaptivity’ has been investigated in species highlighted in blue. NE, no evidence; PR, protection; TgIP, transgenerational immune priming. This figure was created using the following references - 1 González-tokman et al., 2010; 2 McNamara et al., 2014; 3 Faulhaber and Karp, 1992; 4 Cole et al., 2020.; 5 Rosengaus et al., 1999; 6 Vorburger et al., 2008; 7 Schwarz and Evans, 2013; 8 Hernández López et al., 2014; 9 Roth et al., 2009; 10 Salmela et al., 2015; 11 Riessberger-Gallé et al., 2015; 12 Sadd and Schmid-Hempel, 2007; 13 Hamilton et al., 2011; 14 Konrad et al., 2012; 15 Gálvez and Chapuisat, 2014; 16 Reber and Chapuisat, 2012; 17 Rosengaus et al., 2013; 18 Futo et al., 2017; 19 Roth et al., 2010; 20 Tate and Graham, 2015; 21 Eggert et al., 2014; 22 Futo et al., 2015; 23 Knorr et al., 2015; 24 Thomas and Rudolf, 2010; 25 Fisher and Hajek, 2015; 26 Dubuffet et al., 2015; 27 Zanchi et al., 2011; 28 Moreau et al., 2012; 29 Wu et al., 2015a; 30 Miyashita et al., 2015; 31 Wu et al., 2015b; 32 Wu et al., 2015c; 33 Freitak et al., 2014; 34 Trauer-Kizilelma and Hilker, 2015; 35 Trauer and Hilker, 2013; 36 Tidbury et al., 2011; 37 Freitak et al., 2009; 38 Rahman et al., 2004; 39 Mahbubur Rahman et al., 2007; 40 Ma et al., 2005; 41 Apidianakis et al., 2005; 42 Christofi and Apidianakis, 2013; 43 Cabrera et al., 2023; 44 Pham et al., 2007; 45 Mondotte et al., 2018; 46 Mondotte et al., 2020; 47 Linder and Promislow, 2009; 48 Longdon et al., 2013; 49 Moreno-García et al., 2015; 50 Vargas et al., 2020; 51 Rodriguez-Andres et al., 2024; 52 Rodrigues et al., 2010; 53 Ramirez et al., 2015, 54 Contreras-Garduño et al., 2015, 55 Bruner-Montero et al., 2023; 56 Patel and Oliver, 2024. This figure was created using BioRender.com.

The ability of the insect immune system to develop some form of acquired immunity has been documented in a wide range of species (Cooper and Eleftherianos, 2017; Kurtz and Franz, 2003; Trauer-Kizilelma and Hilker, 2015; Freitak et al., 2009; Figure 3). However, the experimental designs used in these studies differ significantly in several key aspects. These include the identity of the pathogen (bacteria, viruses, parasites, fungi), the pathogen dose, the use of homologous (the identical pathogen used twice) versus heterologous (a different pathogen used for the first and second exposure) challenges, the developmental stage of the host, and the interval between the first and second pathogen encounters. In the following paragraphs, we aim to focus mainly on documented cases of immune memory in insects. However, the design of some studies does not allow us to clearly distinguish between different types of acquired immunity.

To assess the ability to develop specific immune memory and distinguish it from other types of acquired immunity, we suggest following several key principles when designing an infection assay. First, the pathogen used in both the first and second challenges must be identical (homologous infection). To distinguish immune memory from immune training, a different pathogen should also be used for the second challenge. If the second challenge results in improved resistance to this different pathogen, it suggests immune training rather than memory. The nature of the pathogen itself should also be considered as Gram-positive and Gram-negative bacteria, fungi, and viruses activate different immune pathways (Wang et al., 2010; Cheng et al., 2016). Additionally, the infection dose and route should be carefully chosen as they can influence the activation of downstream signaling pathways and determine which components of the immune system are engaged (Pereira et al., 2024). It is also important to consider whether the pathogen is naturally associated with the insect species in question as some species may exhibit high tolerance to certain pathogens. Tolerance refers to the ability of an organism to limit the negative fitness consequences of infection without necessarily reducing the pathogen load (Schneider, 2021). Even the genetic identity of the pathogen can be critical as different strains may vary in antigenicity or induce cross-reactive immunity (Johnson et al., 2012; Roth et al., 2009). Crucially, the interval between the first and second doses must be long enough to allow for the complete clearance of the pathogen from the organism and to give the immune system time to develop immune memory. If the pathogen persists, the immune system may remain in an activated state, making it difficult to determine whether a true memory response or simple immune priming is occurring. Therefore, immune activation readouts should return to baseline levels before administering the second dose as immune memory functions on biphasic principles.

In the following paragraphs, we focus on documented cases of acquired immunity with emphasis on immune memory, followed by documented cases of acquired immunity across life stages and across generations. We begin with *D. melanogaster*, the most tractable insect model organism (Arch et al., 2022).

The first study to document the existence of some form of immune adaptivity in *D. melanogaster* was published in 2005. In this study, the authors demonstrated that flies that were first inoculated with an avirulent strain of *Pseudomonas aeruginosa* mount an increased resistance to a highly virulent strain. However, the second bacterial dose was administered no later than 24 hours after the initial exposure (Apidianakis et al., 2005). While there is no doubt that survival rates improved, this may be attributed to immune priming rather than immune memory, given the extremely short time window between exposure to the low-virulence and virulent strains. This suggests that immune activation did not have sufficient time to return to baseline. In a follow-up study, the time interval between doses was extended to 11 days; however, by day 10, the bacteria were still present in the system, which again suggests priming rather than immune memory. Similar priming effects have also been observed in numerous other studies, achieved through both systemic and oral administration (Christofi and Apidianakis, 2013; Cabrera et al., 2023).

The study by Pham and colleagues may have more testimonial value regarding immune memory in *D. melanogaster*. Flies primed with either a sublethal dose or heat-killed *Streptococcus pneumoniae* were protected against a lethal challenge of the same bacterium administered 7 days later, allowing sufficient time for pathogen clearance. Since this protection was not observed against other selected pathogens, it suggests a highly specific immune response rather than a general activation of antibacterial immunity. A similar effect was achieved by administering a sublethal dose of the natural fungal pathogen *Beauveria bassiana*, which provided protection against a subsequent lethal dose of the same fungus. However, the ability to develop protection against future pathogen encounters may not be universal for all pathogens as administration of heat-killed *Salmonella typhimurium*, *Listeria monocytogenes*, and *Mycobacterium marinum* did not confer protection against subsequent lethal challenges with these respective bacteria (Pham et al., 2007).

Many studies on insect immune memory have also been conducted using models other than *D. melanogaster* (Freitak et al., 2009; Wang et al., 2010; Cheng et al., 2016; Pereira et al., 2024). A remarkably high degree of specificity of immune memory has been documented in *Tribolium castaneum*, whose immune system can discriminate between closely related bacterial strains of *Bacillus thuringiensis* (Roth et al., 2009; Futo et al., 2017). Narrow specificity and increased protection have also been observed in the bumblebee, *Bombus terrestris*, where the immune memory lasts for several weeks after clearance of bacteria from the first exposure (Sadd and Schmid-Hempel, 2006).

Interestingly, immune memory in insects may potentially persist across different life stages. Mondotte and colleagues conducted trans-stadial assays, revealing that adult flies orally infected with *Drosophila C virus* during the larval stage exhibited increased tolerance to subsequent reinfections with the same virus. Furthermore, when adult flies derived from larvae infected with *Drosophila C virus* were reinfected with different viruses, their survival rates were comparable to those of the control group. This indicates that the acquired protection is both virus-specific and sequence-specific (Mondotte et al., 2018). Trans-stadial acquired immunity has also been documented in *Aedes aegypti,* where infection of larvae with inactive dengue virus has been shown to protect against subsequent infection in adult mosquitoes (Vargas et al., 2020). Similar protective effects in adults have also been observed following exposure of *A. aegypti* larvae to *Escherichia coli* (Moreno-García et al., 2015). Trans-stadial protection has also been documented in another mosquito, *Anopheles arabiensis*. In this study, larval exposure to *Streptococcus pyogenes* or *E. coli* led to increased longevity of adult females challenged with the respective pathogen (Patel and Oliver, 2024). An intriguing model for investigating acquired trans-stadial immunity during host–parasite interaction is *Tribolium confusum* infected with the parasite *Gregarina minuta* because the gut parasites are naturally shed during metamorphosis along with the gut lining. Indeed, this model has shown that adult beetles infested with *G. minuta* display enhanced survival resistance when infected as larvae, highlighting the persistence of immune protection across life stages (Thomas and Rudolf, 2010).

Intriguingly, several studies have documented the ability of insects to pass immune experiences acquired during pathogen exposure to their offspring, a phenomenon known as transgenerational immune priming (TgIP). Since mounting an immune response is connected with significant energy costs, TgIP is likely to evolve in environments where offspring have a high probability of encountering the same pathogen as their parents (Thomas and Rudolf, 2010). This is especially plausible in social insects, where worker offspring remain in the natal nest. In 2014, a study was published in which researchers challenged honeybee queens by injecting a vegetative form of heat-killed *Paenibacillus larvae*, the causative agent of American foulbrood (Genersch, 2010). Although adult honeybees are resistant to *P. larvae* infection, they act as vectors, transmitting the disease to the brood (Fries et al., 2006). The larvae of immunized queens were subsequently fed a diet containing *P. larvae* spores, that is, the infectious stage. It has been shown that these individuals display improved survival compared to larval progeny of nonimmunized queens. This enhanced survival was presumably due to a threefold increase in the number of differential hemocytes (all hemocyte types except for prohemocytes) (Hernández López et al., 2014). TgIP has also been repeatedly documented in another hymenopteran species, the bumblebee (*B. terrestris).* When queens were challenged with heat-killed *Arthrobacter globiformis*, both their eggs and progeny workers exhibited significantly higher antibacterial activity when injected by lipopolysaccharides, compared to controls (Sadd and Schmid-Hempel, 2007). However, such TgIP has been connected with certain costs since these individuals were more susceptible to a pathogen distinct from maternal challenge, the trypanosome parasite *Crithidia bombi* (Sadd and Schmid-Hempel, 2007). The costs of TgIP have also been investigated in tobacco hornworm *Manduca sexta*, where female offspring of immunized parents laid fewer eggs than those derived from control parents (Trauer and Hilker, 2013)*.*

Female TgIP is in agreement with parental investment theory, according to which females invest more into rearing their offspring than males. Nonetheless, as evidenced by research conducted on the red flour beetle *T. castaneum*, TgIP is not restricted to maternal effects. After parental exposure to heat-killed *E. coli* or *B. thuringiensis*, TgIP occurs through fathers as well as mothers (Roth et al., 2010). Similar results were obtained in another coleopteran species, the yellow mealworm beetle *Tenebrio molitor*, where adult males and females were immune-challenged with lipopolysaccharide (LPS), and their progeny showed improved response to LPS. However, the underlying mechanism of TgIP in mothers and fathers seems to be different as stimulation of different immune effectors in the offspring has been recorded, and the duration of the protection also differs according to the sex of the challenged parent. While the offspring of LPS-treated mothers displayed an increased number of hemocytes and unaffected phenoloxidase and antibacterial activity before and after immune stimulation, offspring males showed no alterations in the number of hemocytes or phenoloxidase and antibacterial activity, but they displayed the ability to develop a stronger immune response mediated by the prophenoloxidase. Nonetheless, these effects were observed only in offsprings laid within the first 4 days after paternal challenge (Zanchi et al., 2011). A study published by Patel and Oliver suggests that TgIP in *An. arabiensis* may be influenced by the insecticide-resistant phenotype as protection was observed in insecticide-susceptible *An. arabiensis* but not in their resistant counterparts (Patel and Oliver, 2024).

Evidence of TgIP has also been found against viral pathogens. Offspring of Indian meal moth (*Plodia interpunctella*) parents exposed to a low dose of their natural DNA virus during the larval stage were less susceptible to viral challenge (Tidbury et al., 2011). Further insights into TgIP were provided by a study published by Mondotte and colleagues, which clearly demonstrated that the acquired immune protection is both virus- and sequence-specific, persisting across generations. Using *D. melanogaster* and *A. aegypti* as models, the authors revealed that the offspring of immune-challenged parents inherit viral DNA and exhibit upregulation of genes related to chromatin and DNA binding (Mondotte et al., 2020). Similar results were obtained in a recent study by Rodriguez-Andres and colleagues, who documented that *A. aegypti* mosquitoes infected with arboviruses can transmit specific immunity to their offspring (Rodriguez-Andres et al., 2024). Additionally, alterations in the epigenetic landscape, specifically DNA methylation and histone acetylation, were detected in the F1 generation of parents fed non-pathogenic *E. coli* or the entomopathogen *Serratia entomophila* (Gegner et al., 2019). These findings highlight the role of epigenetic mechanisms in TgIP.

### Mechanisms of insect immune memory

In the preceding paragraphs, we presented experimental evidence demonstrating that immune memory occurs across a wide range of insect species, spanning all major insect orders. Moreover, immune memory appears to persist through developmental stages and may even be passed on to subsequent generations. To explore the potential mechanisms underlying immune memory formation in insects, we can draw inspiration from the previously well-characterized processes of antiviral adaptive immunity in prokaryotes and adaptive immunity in mammals, and infer general principles that any immune memory mechanism must follow. However, the particular components of antiviral and antibacterial immune memory likely differ significantly as CRISPR-based immunity has not been identified in any eukaryotes and insects lack T- and B-lymphocytes, as well as the genes coding for antibodies.

### Acquired immune memory can be in general divided into three phases

#### Antigen acquisition

During the initial encounter with a pathogen, pathogen-specific antigenic molecules must be captured and distributed to other immune cells throughout the body.

#### Development of immune specificity

These acquired antigens serve as templates for the formation of antigen- or sequence-specific immune memory.

#### Maintenance of immune memory

The immune system retains these molecules to enable the efficient and specific recognition of the pathogen in future encounters, ensuring its rapid elimination.

### Insect’s adaptive antiviral immunity

Insects frequently face infections from various DNA and RNA viruses (Bruner-Montero et al., 2023). The life cycle of insect viruses does not differ significantly from that of viruses infecting other eukaryotes. Upon entry into a host cell, viruses hijack the host’s transcription and translation machinery with the goal of replicating their genetic material and synthesizing proteins for the capsid, facilitating further viral propagation (Wang et al., 2024). Once a cell is infected, it activates several cell-autonomous antiviral immune mechanisms, relying primarily on RNAi, stalled translation, nucleotide-based antiviral strategies, induction of autophagy, and ultimately, apoptosis (Tafesh-Edwards and Eleftherianos, 2020). Although these antiviral strategies significantly reduce virus replication in infected cells, they are not sufficient to make individuals fully resistant to viral infection.

Recent discoveries have shown that the systemic immune response is critical for host resistance to viral infections in *Drosophila* (Kingsolver et al., 2013). Virus-infected cells commonly activate the transcription factors Hop and Dome, leading to increased production of unpaired family cytokines (*Upd1*, *Upd2*, *Upd3*). Elevated levels of these cytokines in circulation activate macrophage-like plasmatocytes via the JAK-STAT signaling pathway, attracting them to virus-infected tissues (Sabin et al., 2010). The elimination of virus-infected apoptotic cells by activated plasmatocytes has been shown to be crucial for viral resistance in *Drosophila* (Saleh et al., 2009). However, plasmatocytes do not simply degrade all engulfed material; the presence of viral double-stranded RNA in the phagolysosome leads to permeabilization of its membrane and acquisition of viral genetic information (Tanaka et al., 2024). Plasmatocytes then use viral RNA as a template for reverse transcription, integrating fragments of complementary antisense viral DNA into their genomes (Tassetto et al., 2017). Since eukaryotic cells lack the enzymes necessary for these processes, they are facilitated by the activity of domesticated retrotransposons. The exact mechanism by which retrotransposons are engaged during infection has not yet been clarified. Nevertheless, retrotransposon activity increases more than 20-fold in plasmatocytes upon viral infection, and mere inhibition of retrotransposons completely abolishes the host’s resistance to infection. Sequencing of circular DNA from infected plasmatocytes reveals that virus-derived DNA is commonly flanked by sequences from Ty2 and Ty3 retrotransposons of the LTR family (Roy et al., 2020).

**Figure 4.**
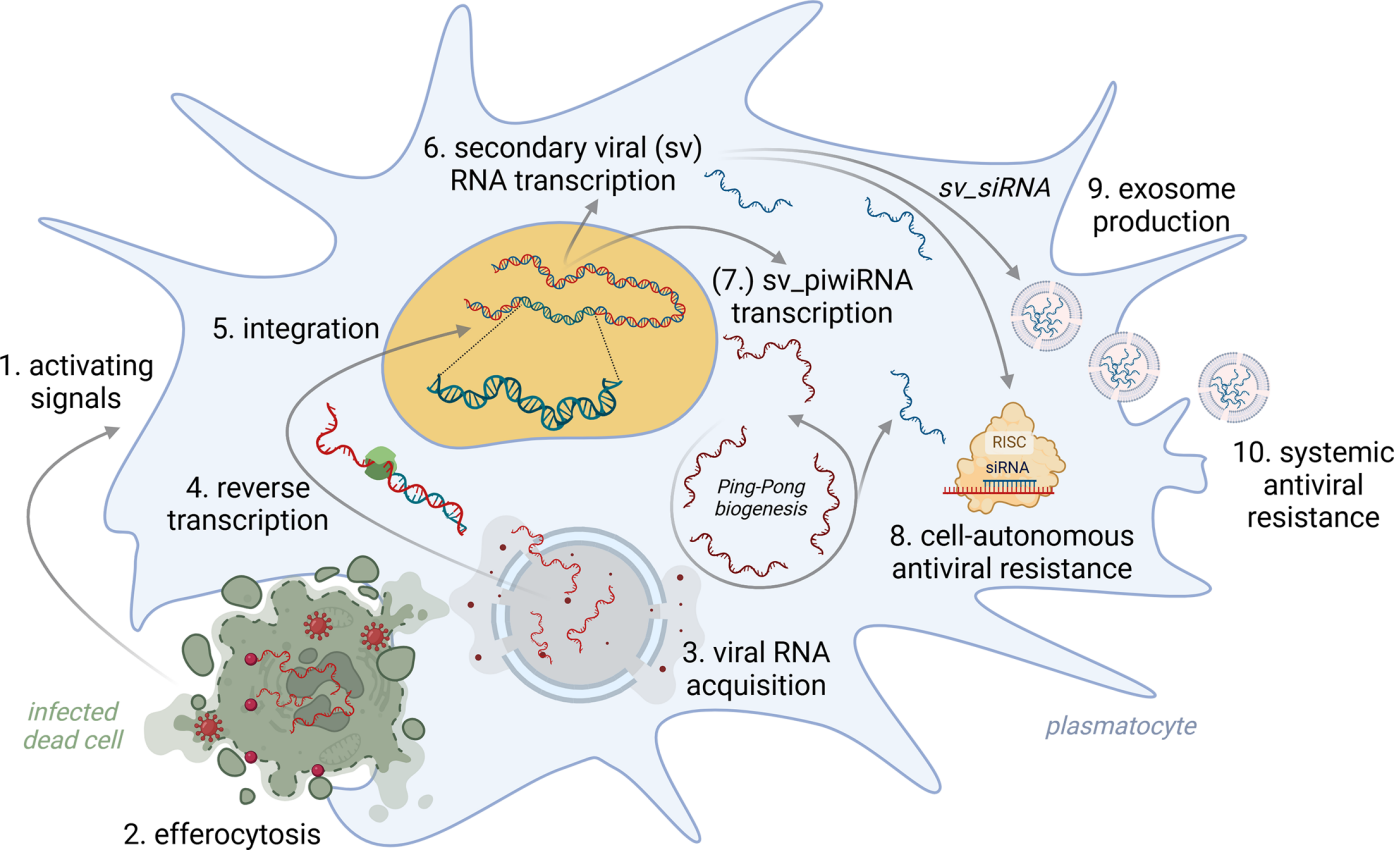
Schematic representation of the hypothetical mechanism underlying the formation of antiviral immune memory in insects. Virus-infected cells produce signals that attract macrophage-like plasmatocytes (1), which eliminate them through a process known as efferocytosis (2). The engulfed cellular debris, along with viral particles, is degraded in the phagolysosome, where viral RNA is acquired (3). This viral RNA is then reverse transcribed (4) and integrated into the genome of host plasmatocytes through the activity of co-opted retrotransposons (5). The secondary viral RNA produced (6) can either undergo amplification in the cytosol via the ping-pong biogenesis mechanism (7) and serve as a template for sequence-specific cellular immunity mediated by the RNA interference (RNAi) pathway (8) or be packaged into exosomes for distribution to other cells within the organism (9). Through this mechanism, plasmatocytes provide systemic antiviral immunity to tissues and cells before they encounter the virus (10), while also retaining imprints of past viral infections in the form of integrated viral sequences. This figure was created using BioRender.com.

Viral RNA, integrated into specific regions of the host genome as endogenous viral element (EVE), serves as a template for producing secondary viral DNAs (svDNAs) (Tassetto et al., 2017). Expression of svDNAs subsequently provides cells with cell-autonomous resistance to viral infection as it primes the activation of *Dicer* and *Argonaute* proteins, which together form the RISC that governs sequence-specific elimination of viral RNA (van Rij et al., 2006). Additionally, plasmatocytes disseminate svDNA systemically, enclosed within extracellular vesicles or via tunneling nanotubes, along with activating cytokine signaling (Tassetto et al., 2017). Thus, plasmatocytes, even without being infected themselves, can amplify viral DNA and spread it as a form of prophylaxis to other cells, enabling them to acquire resistance against specific viruses (Mondotte et al., 2020; Figure 4).

Similar to the CRISPR-Cas system, insect antiviral immune memory is based on the integration of viral sequences into the genome, which is later used for sequence-specific elimination of viral nucleic acids. Notably, the integration of viral DNA into the host genome also provides insects with transgenerational adaptive immunity (Mondotte et al., 2020; Rodriguez-Andres et al., 2024).

Despite certain variations in the molecular mechanisms, this general process appears to be widespread among insects. Comparisons of small RNAs produced from EVE regions in different insect species indicate that analogous mechanisms likely occur in ants (Hymenoptera), beetles (Coleoptera), aphids (Hemiptera), butterflies (Lepidoptera), mosquitoes and flies (Diptera), and even ticks (Ixodida, Arachnida, Chelicerata) (Gilbert and Belliardo, 2022). Recently, an analogous mechanism has also been identified in crustaceans, suggesting that the mechanism of antiviral immune memory may be common across most arthropods (Taengchaiyaphum et al., 2021).

In some of these species, EVE transcription gives rise not only to siRNA but also to longer transcripts known as Piwi-interacting RNAs (piRNAs). piRNAs bind to RISC complex proteins, Piwi (PIWI) and Aubergine (AUB), and enter the secondary piRNA biogenesis pathway in the cytosol, also known as the ‘ping-pong’ pathway (Tassetto et al., 2019). This allows the cell to amplify the signal and produce a much larger quantity of protective RNA fragments, which confer viral resistance and spread this resistance to other cells. This mechanism may provide mosquitoes with a heightened ability to resist viral infections without displaying any obvious pathological phenotypes. This aligns with the persistence of viral infections in these species, which is associated with their capacity to transmit a variety of viral diseases (Trammell and Goodman, 2021).

Evidence of sequence-based adaptive immune memory in insects raises the question of whether an analogous antiviral strategy may also occur also in mammals. Although experimental evidence for such a mechanism is currently lacking, there are indications that a similar mechanism might exist in mammals but is overshadowed by other dominant antiviral immune systems. Recent findings highlight the importance of cellular immunity, specifically the RNAi cascade, in antiviral defense mechanisms (Berkhout, 2018). Studies have documented that in mice viral infections are accompanied by the systemic spread of virus-derived small interfering RNAs via extracellular vesicles, which provide recipient cells with acquired resistance to subsequent viral infections (Zhang et al., 2022). However, contradictory findings in mammals suggest that *Dicer* exhibits negligible enzymatic activity in virus-infected cells, and its knockdown does not substantially affect viral load (Berkhout, 2018; Wang and Li, 2024). This phenomenon certainly warrants further investigation.

### Insect’s adaptive antibacterial immunity

While the mechanism of antiviral immune memory formation is relatively well understood, the mechanisms underlying antibacterial immune memory remain more elusive. Nevertheless, we can assume that the formation of antibacterial immune memory would still involve phases of antigen acquisition, development of specificity, and memory maintenance.

Once bacteria breach primary immune barriers, they are recognized by professional phagocytes, which engulf and eliminate them in the phagolysosome. In contrast to mammals, where antigen acquisition from the phagolysosome of macrophages is well understood, this process remains largely unexplored in insects (Hultmark and Borge-Renberg, 2007). Although insects lack the *major histocompatibility complex* (MHC) and *tapasin* (Tap) genes, essential for canonical antigen presentation in mammals, certain evidence suggests that other components of the antigen processing machinery may be conserved. This is demonstrated by the fact that simply expressing these two genes heterogously in *Drosophila* S2 cells, derived from embryonic macrophages-like cells, enables them to present antigens on their surface (Ortmann et al., 1997). Therefore, we may assume that insect macrophage-like plasmatocytes process antigens similarly to their mammalian counterparts and present them to other immune cells in the body through a noncanonical pathway. Recent studies in mice have shown that mammalian macrophages disseminate antigens via extracellular vesicles, encapsulated within multilamellar bodies, or through a process known as eruptophagy, where the phagolysosome’s contents are released directly into the extracellular space (Smith et al., 2017; El Safadi et al., 2024; Lindenbergh and Stoorvogel, 2018). It is plausible that these primitive methods of antigen dissemination represent ancestral forms, with the addition of tapasin and MHC complexes evolving later alongside vertebrate adaptive immunity (Kaufman, 2018). This idea aligns with numerous observations in insects, where circulating bacterial antigens have been found in complexes with hemolymph proteins such as lipoproteins, vitellogenins, and several storage proteins (Salmela et al., 2015; Kato et al., 1994; Kanost and Zhao, 1996). However, further research on antigen processing and dissemination in insects is necessary.

**Figure 5.**
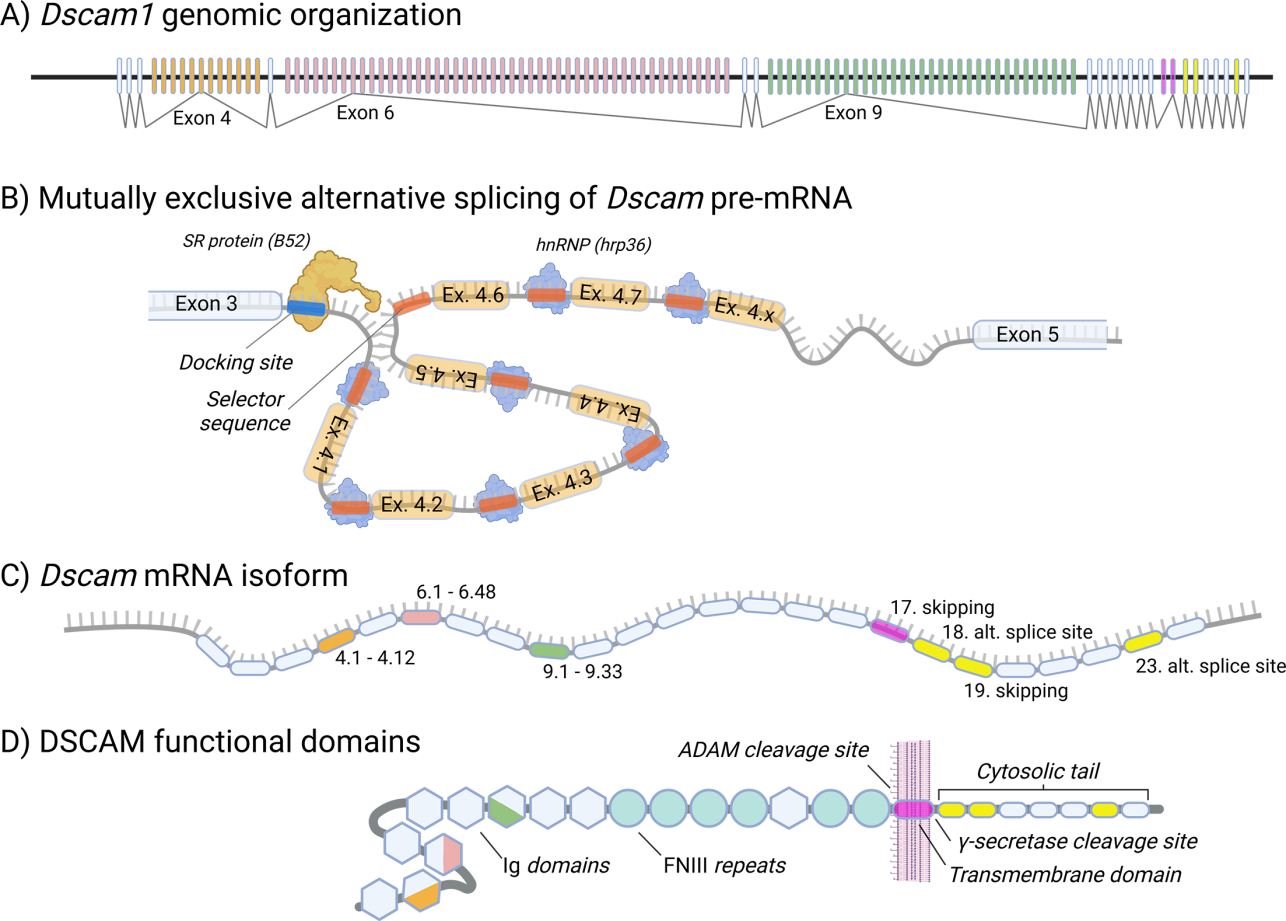
Structure and variability of the *Drosophila Dscam1* gene. (**A**) Schematic representation of the *Dscam1* genomic region, highlighting the hypervariable exons 4, 6, and 9. (**B**) Diversity in the immunoglobulin domains of *Dscam1* arises through mutually exclusive alternative splicing of these hypervariable exons, exemplified here by exon 4. This splicing process is tightly regulated by serine/arginine-rich (SR) proteins and heterogeneous nuclear ribonucleoproteins (hnRNPs). (**C**) A single *Dscam1* gene can generate thousands of isoforms, which may exist as full-length, membrane-bound proteins or as truncated, soluble variants circulating in the hemolymph. (**D**) The DSCAM protein structure consists of 10 immunoglobulin domains, 6 fibronectin type III (FNIII) repeats, a transmembrane domain, and a cytoplasmic tail involved in signaling and regulatory functions. This figure was created using BioRender.com.

A crucial step in forming immune memory is the recognition of pathogen surface antigens, similar to how multivariant immunoglobulin domains of antibodies function in vertebrates. Insects possess numerous genes containing immunoglobulin domains capable of binding bacterial antigens, among which the gene coding for *Down syndrome cell adhesion molecule* (*Dscam*) is particularly noteworthy (Watson et al., 2005). *Dscam* consists of extracellular, transmembrane, and cytosolic regions. The extracellular part of *Dscam* contains 10 immunoglobulin domains and 6 fibronectin type III repeats (Wang et al., 2012). Three of the immunoglobulin domains (2, 3, and 7) are encoded by multivariant exons (4, 6, and 9), which, in *Drosophila*, have 12, 48, and 33 alternative variants, respectively (Wojtowicz et al., 2007). While the general structure of *Dscam* is conserved across arthropods, the number of multivariant exons varies significantly among species (Ng and Kurtz, 2020). Individual DSCAM variants are produced through mutually exclusive alternative splicing, which ensures that only one variant from each multivariant exon is included in the final mRNA (Graveley, 2005). Consequently, *Dscam* can generate up to 19,008 (12 × 48 × 33) immunoglobulin domain variants, with additional variability arising from alternative splicing of exon 17, producing either membrane-bound or secreted forms (Celotto and Graveley, 2001; Figure 5).

*Drosophila Dscam* is the gene with the highest number of alternative transcripts in animals, drawing significant scientific attention to the mechanisms regulating such a complex process. Inclusion of a single variant from a multivariant exon is achieved through the pairing of a ‘docking sequence’ at the end of the previous exon with a ‘selector sequence’ preceding each variant in the specific multivariable exon (Graveley, 2005). While the docking sequence interacts with a serine-arginine protein (SR-protein) to initiate the inclusion of the following exon, selector sequences are occupied by heterogeneous nuclear ribonucleoproteins (hnRNPs), which inhibit the inclusion of specifically occupied variants through steric hindrance (Olson et al., 2007). This process is further complicated by the influence of intronic RNA secondary structures called ‘inclusion RNA stem-loops’ (iStems) (Kreahling and Graveley, 2005). Moreover, it seems that each of the three multivariant exons shows specific characteristics in the alternative splicing mechanism (Ustaoglu et al., 2019; McManus and Graveley, 2011), which aligns with recent identification of more than twelve RNA-binding proteins involved in *Dscam* alternative splicing in S2 cells (Brooks et al., 2015).

In addition to the variability of *Dscam* in its immunoglobulin domains, there is also diversity in its cytosolic tail. It has been reported that alternative exon skipping and selection of alternative splicing sites for exons 18 and 23 generate eight different variants of *Dscam* cytosolic tail. This diversity may play an important role in intracellular *Dscam* signaling, a topic to be discussed later; however, no experimental data are currently available on this aspect (Sachse et al., 2019).

*Dscam* plays a dual role in insects; in addition to its function in immune response, it is essential for the development of the central nervous system (CNS) (Schmucker et al., 2000). The combination of several *Dscam* splicing variants provides each neuron in the *Drosophila* CNS with a unique recognition code (Hattori et al., 2007). Each DSCAM variant can form homodimeric bonds with another DSCAM molecule containing identical domains, allowing neurons to recognize their own cellular surfaces. This homodimeric interaction triggers intracellular signaling, leading to the remodeling of the actin cytoskeleton and repulsion of cellular extensions (Wojtowicz et al., 2004). This process prevents neurons from forming self-stimulating loops as they create complex structures in the developing CNS.

The role of *Dscam* in immune responses to bacterial pathogens is of particular interest. It has been shown that *Dscam* is highly expressed in *Drosophila* plasmatocytes during bacterial infection (Celotto and Graveley, 2001). Both circulating and membrane-bound forms of DSCAM are produced by plasmatocytes, facilitating the opsonization and phagocytosis of pathogens (Watson et al., 2005). It is believed that the binding of DSCAM to a pathogen alters its tertiary structure, allowing it to be recognized by membrane-bound DSCAM, which facilitates internalization through phagocytosis (Dong et al., 2006; Wan et al., 2023). It remains unclear whether each DSCAM variant has its own receptor and whether internalization requires homodimeric binding, or whether a general membrane-bound DSCAM can mediate internalization of any DSCAM variant. However, both circulating and membrane-bound forms of DSCAM are necessary for combating bacterial infections (Li, 2021; Li et al., 2019).

During immune responses, *Dscam* undergoes alternative splicing, enabling plasmatocytes to produce nearly the full spectrum of DSCAM variants. However, this alternative splicing is not a random process in plasmatocytes. Infection by a specific pathogen results in the selection of specific *Dscam* variants that exhibit particularly high affinity for the pathogen’s surface antigens (Meijers et al., 2007). This suggests the presence of a feedback loop that informs the cell which DSCAM variant binds to the pathogen’s surface and should thus be preferentially produced through alternative splicing of *Dscam* pre-mRNA.

Recently, significant progress has been made in understanding the role of *Dscam* in the immune response in crustaceans, leading to the proposal of a potential feedback mechanism. Activation of macrophage-like hemocytes in the Chinese mitten crab (*Eriocheir sinensis*) enhances the production and diversification of DSCAM variants in both circulating and membrane-bound forms (Li et al., 2018). DSCAM that can bind to pathogens is subsequently internalized through homodimeric interaction between circulating and membrane-bound forms, activating the adaptor protein *Dreadlocks* (*Dock*) (Li and Guan, 2004). Dock physically interacts with and activates various serine/threonine kinases, thus triggering signaling events such as phosphorylation and nuclear translocation of the NFĸB homolog Dorsal, as well as the activation of MAPK-ERK-JNK signaling cascade. These signals lead to increased production of antimicrobial peptides and a shift in the ratio of hnRNP (*hrp36*) and SR-protein (*B52*). This shift may stabilize the alternative splicing machinery and limit *Dscam* splicing to produce only a few preselected DSCAM isoforms (Li et al., 2019). However, it remains unclear if a simple change in the HRP36 and B52 ratio is sufficient for stabilizing *Dscam* alternative splicing. In *Drosophila*, genetic manipulation of HRP36 and B52 strongly influences *Dscam* alternative splicing in the CNS, but it tends to cause the inclusion of multiple multivariant domains from a single exon, resulting in dysfunctional DSCAM (Olson et al., 2007). However, the situation in activated hemocytes may differ significantly from that in the CNS as the variability induced by infection is not developmentally regulated.

In addition to stabilizing splicing, *Dscam* signaling further activates the *a disintegrin and metalloproteinase*, which sheds the outer DSCAM domains, enabling cleavage of its cytosolic domain by γ-secretase (Li et al., 2021). The intracellular domain then interacts with importin proteins, which guide it to the nucleus, initiating a program that promotes cell persistence and proliferation (Xia et al., 2024; Ng et al., 2014). The signaling mechanism of the cytosolic tail is not fully understood, and given that eight different variants have been observed in cells, it may be relatively complex. However, whether alternative splicing of the cytosolic domain contributes to immune memory remains unknown. Shedding of the extracellular domain may signal that the cell no longer needs to actively fight the pathogen, and this, coupled with increased persistence and proliferation, could provide a basis for the development and maintenance of immune memory. Although the main components of the described *Dscam* signaling pathway are known to play roles in the *Drosophila* CNS (Sachse et al., 2019), their implementation and significance in immune-related processes in insects remain to be addressed.

This brings us to the last stage of immune memory formation, which is the maintenance of preselected DSCAM variants for a secondary encounter with the pathogen. However, experimental data documenting this process are currently lacking, leaving the underlying mechanism open to speculation.

It has already been mentioned that cells expressing preselected DSCAM variants may proliferate, thus increasing their numbers (Xia et al., 2024). Indeed, infection-induced proliferation is commonly observed in many insect species, particularly during larval and pupal stages (Eleftherianos et al., 2021). However, some insect species with well-documented immune memory display limited proliferative potential in immune cells during the adult stage, and no specific hematopoietic organs or niches have been identified as responsible for immune memory maintenance so far (Sanchez Bosch et al., 2019; Ghosh et al., 2015).

An alternative explanation could involve the storage of preselected DSCAM variants in the form of either mRNA or translated proteins. *Dscam* mRNA might be stored in various membraneless organelles, such as p-bodies, or ω-speckles (Luo et al., 2018), consistent with evidence of numerous RNA-binding proteins interacting with *Dscam* mRNA in *Drosophila*. Likewise, preselected DSCAM variants in protein form could accumulate in cytosolic protein granules, bind to the surface membrane of plasmatocytes, or circulate in the hemolymph.

**Figure 6.**
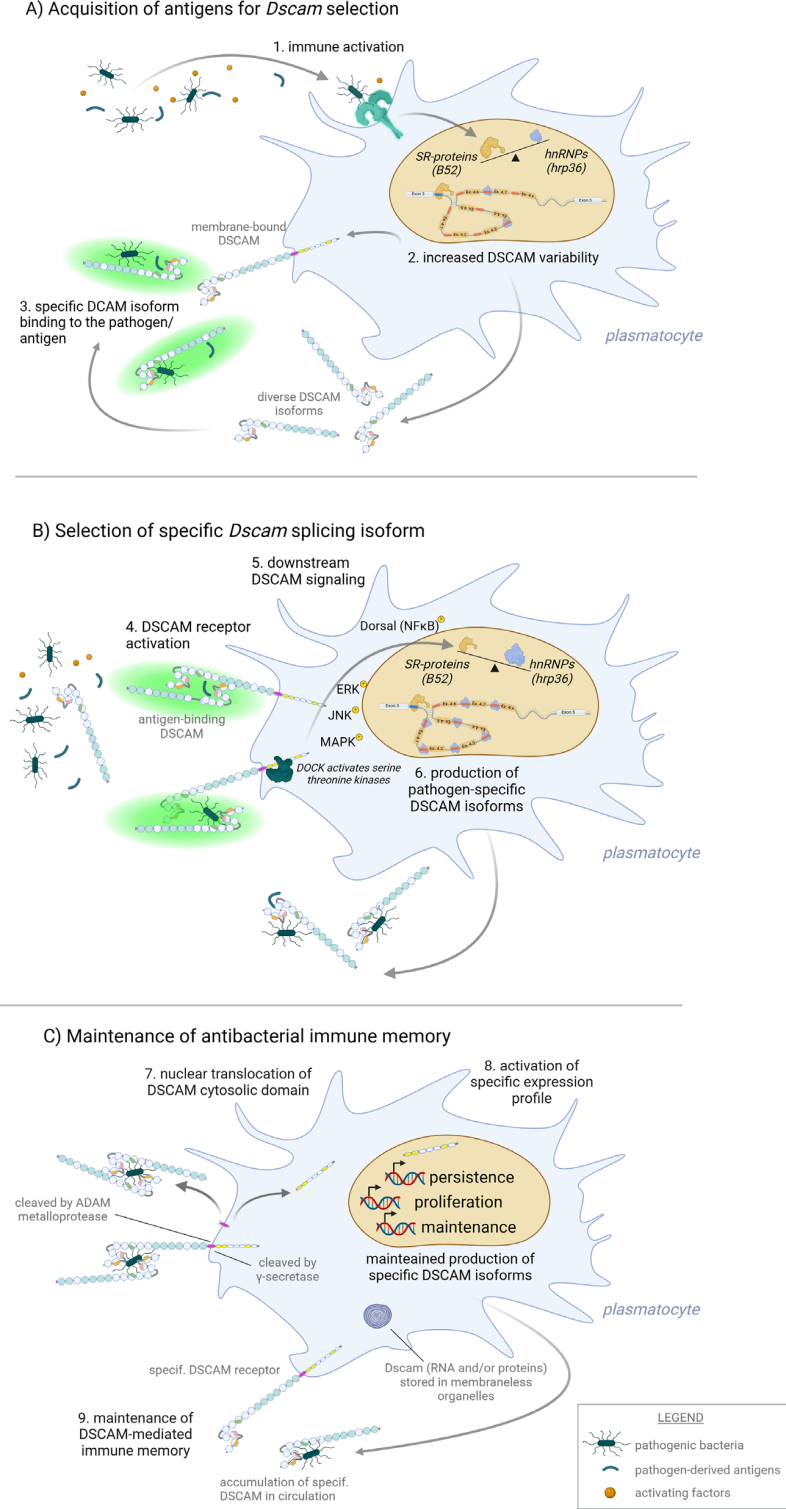
Schematic representation of the hypothetical mechanism underlying the formation of *Dscam*-based antibacterial immune memory in insects. The recognition of pathogenic bacteria by plasmatocytes triggers the activation of key classical immune pathways, leading to the production of humoral immune factors, increased cell motility, and metabolic polarization (1). Additionally, changes in epigenetic regulators, including serine-arginine protein (SR-protein) and heterogeneous nuclear ribonucleoproteins (hnRNPs), induce high variability in the alternative splicing of the *Dscam* gene, resulting in the production of a diverse repertoire of DSCAM molecules (2). DSCAM proteins are produced both as circulating opsonization factors and as membrane-bound receptors (3). When a specific DSCAM variant exhibits high affinity for pathogen surface antigens, signaling through the DSCAM receptor is initiated via serine/threonine kinases (MAPK, JNK, ERK). (5). This leads to the stabilization of alternative splicing, ensuring the production of only DSCAM variants that specifically recognize the pathogen (6). Subsequently, the DSCAM receptor undergoes proteolytic cleavage by ADAM metalloproteases and γ-secretase, releasing its cytosolic domain (7). This cleaved cytosolic domain translocates to the nucleus, where it directly participates in the regulation of gene transcription involved in cell persistence, maintenance, and proliferation (8). These processes contribute to the long-term persistence of immune-activated cells and the maintenance of immune memory (9). While a significant portion of this hypothetical model is supported by robust scientific data, further experimental validation is required to confirm its mechanisms. This figure was created using BioRender.com.

Anchoring DSCAM receptors to the plasmatocyte membrane may increase the immune system’s sensitivity to the pathogen. Recognizing a pathogen and producing preselected, pathogen-binding DSCAM forms could potentially eliminate the infection in its early stages (Figure 6).

Although *Dscam* signaling plays a crucial role in pathogen recognition and opsonization, other immune responses, such as phagocytosis, the production of ROS, antimicrobial peptides, and melanization, also contribute to pathogen elimination. However, there is no experimental evidence suggesting that these responses are triggered in a pathogen-specific manner. Consequently, they are unlikely to serve as the primary mechanisms of immune memory formation but may function as important complementary processes. For instance, cytokine-like molecules could act as co-stimulatory factors during immune memory formation.

### Might *Drosophila* reveal the mechanism of insect immune memory?

As discussed in the previous section, our understanding of the mechanisms underlying insect immune memory still has considerable gaps. This may be partly due to inconsistencies in infection assays used by different research teams and the ambiguous use of terms like ‘immune priming’, ‘immune training’, and ‘immune memory’ (Divangahi et al., 2021).

A common outcome in studies of insect-acquired immunity is the observation of a stronger and faster immune response that leads to increased resistance to infection. While these findings provide valuable evidence supporting the existence of immune memory in insects, they offer little insights into the mechanisms of antigen acquisition, the formation of specific memory, or the maintenance of specific immunity until a secondary encounter with the pathogen. Research on these specific processes remains limited, leaving many questions about the underlying mechanisms unanswered (Table 1).

**Table 1.** Key questions regarding insect immune memory to be explored in future research.

	Questions to be addressed in future research
**General mechanisms**	Why do certain pathogens induce immune memory formation while others lead to tolerance?
	Are there specific subpopulations of hemocytes dedicated to the formation of immune memory?
	Why does inherited immune memory persist for only a few generations?
	
**Antiviral immune memory**	How is retrotransposon activity controlled upon viral infection?
	What triggers the production of siRNA and piwiRNA from the EVE genomic region?
	How does the cell distinguish between viral nucleic acids and secondary viral nucleic acids?
	How is the infection-activated production of exosomes regulated?
	If antiviral immune memory is inherited, does the incorporation of viral DNA fragments into germ cells occur?
	
**Antibacterial immune memory**	Do individual hemocytes express all DSCAM isoforms, or only a limited subset?
	Is the binding of circulatory DSCAM to membrane-bound DSCAM strictly homodimeric?
	Do circulating DSCAM isoforms bind to other phagocytic receptors?
	How exactly does infection induce Dscam splicing, and how is the pathogen-specific DSCAM isoform stabilized?
	Is the activating signal of membrane-bound, DSCAM isoform transduced into the expression of its soluble form?
	Do hemocytes expressing the pathogen-specific DSCAM isoform undergo expansion?
	Can hemocytes share the pathogen-specific DSCAM isoform to instruct other hemocytes in the body?
	How can antibacterial immune memory be transmitted to progeny?

EVE, endogenous viral element; piRNA, Piwi-interacting RNA; siRNA, small interfering RNA.

Many of these questions may be answered in the future by using the exceptional insect model organism, *D. melanogaster. Drosophila* offers unparalleled genetic tools for manipulating the expression of investigated genes as well as a broad variety of reporter strains (Tolwinski, 2017; Rajan and Perrimon, 2011). The primary strength of *Drosophila* lies in the ability to induce tissue-specific, precisely regulated gene expression using the Gal4-UAS system (Rajan and Perrimon, 2011). This system allows researchers to control gene expression in particular cells, such as immune cells, without affecting other tissues. Furthermore, by combining the Gal4-UAS system with the Gal4 inhibitor Gal80, gene expression can be regulated by external factors, like temperature or hormone administration, in a technique known as a ‘gene switch’ (Duffy, 2002; McClure et al., 2022). This enables researchers to knock down any selected gene exclusively during the primary contact with the pathogen, when immune memory is being formed, without affecting the immune response to a secondary pathogen challenge. Such an approach can help identify genes critical for immune memory formation and provide insights into the underlying mechanisms.

The rapid generation time of *Drosophila* and the availability of thousands of ready-to-use transgenic strains from stock centers make this model especially suitable for unbiased screens to identify unexpected factors that may be essential for understanding immune memory formation and its transgenerational inheritance.

Research on *Drosophila Dscam1* has primarily focused on its role in CNS development. This work has provided fundamental insights into *Dscam* intracellular signaling, variability, and alternative splicing mechanism (Schmucker and Chen, 2009; Liu et al., 2020). In addition, many new tools were developed to facilitate genetic manipulation of *Dscam* splicing and characterize its isoforms. These tools could also be employed in future research on immune memory formation.

The insect immune system shows many evolutionarily conserved features with the mammalian innate immune system. Yet, at first glance, it may seem unlikely that the antiviral and antibacterial immune memory mechanisms identified in *Drosophila* could be conserved in mammals. However, some evidence suggests that antiviral immune memory may be present in some mammals to a degree, though it may be masked by other dominant antiviral pathways (Skirmuntt et al., 2020). Its significance might be revealed in some unusual viral infections. Therefore, further investigation into insect immune memory mechanisms and their potential analogs in mammals is necessary.

## Future directions

Understanding the molecular mechanisms of insect innate immune memory could have far-reaching implications, particularly for advancements in immunology, evolutionary biology, biotechnology, and agriculture. Investigating these mechanisms may help trace the evolution of the immune system across invertebrate species. In addition, since insects evolved over 400 million years ago, their ability to ‘remember’ infections without adaptive immunity suggests that innate immune memory is a fundamental trait that predates the evolution of vertebrate adaptive immune system. Unraveling how insects generate immune memory without B and T cells could shed light on the mechanisms of trained immunity in vertebrates. Additionally, it may deepen our understanding of host–pathogen coevolution, potentially uncovering novel strategies pathogens use to evade the host immune response.

The formation of innate immune memory involves epigenetic modifications, such as DNA methylation and histone acetylation, which can persist long after an infection has cleared. Intriguingly, some studies suggest that immune memory can be passed on to offspring, enabling challenged parents to produce progeny with enhanced immune defenses (Gegner et al., 2019). These findings have profound implications for understanding *rapid adaptation* to environmental pressures, lending support to aspects of *Lamarckian evolution*, in which acquired traits can be transmitted across generations.

The capacity of vertebrates to develop immune resistance has historically been leveraged in the development of vaccination (Saleh et al., 2021). Vaccination remains one of the most significant breakthroughs in immunology, saving millions of lives each year and helping humanity overcome some of the deadliest and most devastating diseases (Riedel, 2005). The discovery of adaptive immunity in prokaryotes and insects suggests that vaccination principles might also be applicable to these organisms. However, this potential has yet to be thoroughly explored.

Based on the mechanism of insect antiviral immune memory described above, it is reasonable to assume that delivering viral dsRNA to macrophages could be sufficient for immunization. Macrophages have the ability to amplify immune signals and disseminate them to other cells within the organism. This is supported by preliminary experiments and empirical data, showing that injection or feeding of viral nucleic acids alone can provide individuals with a baseline level of acquired immunity against secondary infections (Saleh et al., 2009; Tassetto et al., 2017).

In mosquitoes, both feeding and injection of viral RNA increase resistance to secondary viral infections, with protective effects observed even after injecting as few as 10^5^ copies of viral RNA (Romoli et al., 2024). Efficient prophylaxis has been achieved also against entomopathogenic fungi as injection of lipopolysaccharides prior to *Metarhizium anisopliae* exposure confers protection in *T. molitor* (Moret and Siva-Jothy, 2003). An innovative approach has also been developed in honeybees, where improved resistance to viral infections has been documented in individuals fed bacteria heterologously expressing plasmids with viral nucleic acid fragments (Ricigliano et al., 2024). Recently, feeding honeybees with attenuated *P. larvae* has proven to be an effective treatment against American foulbrood (Dickel et al., 2022). Overall, however, these experiments are relatively rare, and the mechanisms underlying their effects often remain unclear.

Honeybee colonies are currently threatened by the widespread transmission of several viral and bacterial pandemic diseases (Parveen et al., 2022). Protecting bee colonies from these pathogens is therefore a critical issue that vaccination could resolve (Dickel et al., 2022).

On the other hand, blood-feeding insects, especially in tropical areas, transmit a number of diseases that claim millions of victims every year (Belluco et al., 2023). The challenge posed by insect vectors of human diseases lies in their high tolerance to human pathogens. Mosquitoes, ticks, tsetse flies, and fleas do not sufficiently suppress the pathogens they carry, making them not only ideal vectors but also reservoirs for pathogen multiplication. Manipulating the immune tolerance of these vectors to the pathogens they transmit is a currently considered strategy to reduce pathogen spread and, consequently, the incidence of the diseases they cause (Lambrechts and Saleh, 2019).

## Concluding remarks

Over the past few decades, a substantial body of experimental evidence has accumulated, demonstrating that insects possess a form of specific immune memory. However, inconsistencies in experimental design have hindered the interpretation of results and their broader implications. In this review, we outline three types of acquired immunity in insects and highlight key principles to consider when designing experiments on innate immune memory. Although the mechanisms underlying immune memory formation in insects remain largely unknown, significant progress has been made, particularly through studies in *Drosophila*. This review summarizes current knowledge on the mechanisms of innate immune memory formation and identifies critical unanswered questions for future research. Understanding these mechanisms could be crucial for comparing the molecular basis of acquired immunity across various invertebrates and drawing parallels with immune processes in mammals and other vertebrates. Furthermore, such insights may inform strategies for artificially inducing innate immune memory in crop pollinators or for activating the immune systems of insect vectors against the pathogens they transmit. Such an approach could potentially reduce pathogen spread and, consequently, the incidence of the diseases they cause.
